# Variation in Gamma-Globin Expression before and after Induction with Hydroxyurea Associated with BCL11A, KLF1 and TAL1

**DOI:** 10.1371/journal.pone.0129431

**Published:** 2015-06-08

**Authors:** Amanda J. Grieco, Henny H. Billett, Nancy S. Green, M. Catherine Driscoll, Eric E. Bouhassira

**Affiliations:** 1 Department of Cell Biology, Albert Einstein College of Medicine, Bronx, New York, United States of America; 2 Division of Hematology, Department of Medicine, Montefiore Medical Center/Albert Einstein College of Medicine, Bronx, New York, United States of America; 3 Division of Pediatric Hematology/Oncology/Stem Cell Transplantation, Department of Pediatrics, Columbia University, New York, New York, United States of America; 4 Department of Pediatrics, Division of Hematology-Oncology, AECOM, Bronx, New York, United States of America; Southern Illinois University School of Medicine, UNITED STATES

## Abstract

The molecular mechanisms governing γ-globin expression in a subset of fetal hemoglobin (α2γ2: HbF) expressing red blood cells (F-cells) and the mechanisms underlying the variability of response to hydroxyurea induced γ-globin expression in the treatment of sickle cell disease are not completely understood. Here we analyzed intra-person clonal populations of basophilic erythroblasts (baso-Es) derived from bone marrow common myeloid progenitors in serum free cultures and report the level of fetal hemoglobin production in F-cells negatively correlates with expression of BCL11A, KLF1 and TAL1. We then examined the effects of hydroxyurea on these three transcription factors and conclude that a successful induction of γ-globin includes a reduction in BCL11A, KLF1 and TAL1 expression. These data suggests that expression changes in this transcription factor network modulate γ-globin expression in F-cells during steady state erythropoiesis and after induction with hydroxyurea.

## Introduction

Understanding the mechanisms which govern γ-globin expression in adult erythropoiesis is important for developing therapeutic targets for the β-hemoglobinopathies [1]. In sickle cell disease, induction of γ-globin expression decreases the formation of sickle polymers in red blood cells under de-oxygenation conditions. An increase in fetal hemoglobin (α2γ2: HbF) to a threshold level of 20% has been associated with a more benign disease course [2, 3].

Hydroxyurea (HU) was the first drug approved by the FDA for clinical use in sickle cell patients to induce HbF [4] after its clinical effectiveness in reducing acute disease complications of painful crises and hospitalizations was demonstrated [5, 6]. The ameliorative effects of HU on sickle cell disease also include improved blood flow in the microcirculation and a decreased transfusion requirement [7]. However, clinical and laboratory response to HU is highly variable [8, 9]. Most specifically, not all patients reach a clinically significant increase in HbF even at maximum tolerated dose [10, 11]. Differences in baseline HbF levels and DNA polymorphisms, including a SNP in the BCL11A gene, have been investigated as predictors of response to HU among patients [11–13]. However, no predictor alone or in combination has been able to fully predict HbF induction levels after HU treatment signifying the involvement of other uncharacterized factors [11, 12, 14].

The molecular mechanism of action of HU on γ-globin induction remains elusive, despite the clinical significance for treatment of sickle cell disease and other β-hemoglobinopathies [15]. Largely, expression studies of the effect of HU in erythroid cells have revealed changes in miRNAs and gene groups involved in metabolism, translation, cell cycle and RBC cytoskeleton [9, 16–20]. Only recently have two transcription factors, BCL11A and SOX6, been shown to decrease in response to HU *in vivo* in sickle cell reticulocytes [18]. By contrast, studies with erythroid progenitors derived *in vitro* from PBMCs of hydroxyurea responsive and non-responsive β-thalassemia patients have shown SOX6 expression is high in responders in the presence of HU [19] while BCL11A remained unchanged between patients. These conflicting results may reflect underlying differences in globin transcription factor networks between the two diseases or differences between the types of erythroid progenitors tested.

BCL11A was discovered through genome-wide association studies to be a powerful modulator of human HbF levels in non-anemic individuals [21–23]. This zinc finger protein has subsequently been shown to bind several regions at the β-globin locus including the locus control region (LCR), a cis-regulatory element involved in long-range interactions with β-like genes to modulate globin expression. Together with several other transcription factors, BCL11A coordinates the repression of γ-globin during definitive erythropoiesis [24–26]. At the proximal promoter of the γ-globin gene in human erythroid cells, BCL11A and SOX6 have been demonstrated to bind cooperatively and facilitate contact with the LCR in favor of silencing γ-globin expression [26]

KLF1 has been shown to directly regulate BCL11A expression [27] and is an essential activator of β-globin expression [28, 29] thought to play a central role in stabilizing long range binding between the promoter of the β-globin gene and the LCR in adult erythroid cells [30]. Individuals with haplo-insufficiency for KLF1 show increased levels of HbF [31].

TAL1 is a core erythroid transcription factor which assembles with GATA1, LMO2 and LDB1 in a multimeric protein complex during erythroid maturation [32–35]. This complex induces globin gene expression by facilitating direct contact between the locus control region and the globin gene promoters via chromatin looping [34, 36, 37].

Expression of HbF in normal adult blood is generally below 1% of total hemoglobin and occurs in a fraction of red blood cells termed F-cells [38, 39]. The origin of these F-cells and determination of cellular heterogeneity in globin gene expression within the same individual are not well understood as erythroblast progeny of the same BFU-E can express variable levels of γ-globin demonstrating clonal variability [40]. Changes in the epigenetic program in erythroid progenitors during definitive erythropoiesis may determine γ-globin expression in the more mature progeny, and a class of early precursors may exist that can produce descendent stem cells with or without commitment to HbF production. Whether the same transcription factors are involved in generating cellular heterogeneity in globin gene expression within the same individual during steady state erythropoiesis, after HU induction and in the variation of HbF expression between individuals is not fully characterized.

Using an approach based on the analysis of clonal populations of basophilic erythroblasts (baso-Es) derived in serum free culture of bone marrow (BM) common myeloid progenitors (CMP), we report here that the level of HbF production in baso-Es is not yet determined at the CMP level since sub-cultures of daughter cells derived from the same CMPs exhibit different levels of γ-globin expression. We also show that the variations in HbF levels in normal basophilic erythroblasts negatively correlates with expression of BCL11A, KLF1 and TAL1 and that the same genes are further down-regulated after successful induction with HU.

## Material and Methods

### Primary Sample Sources

10mL of peripheral blood was obtained from an equal number of male and female sickle cell patients ranging in age from 11–52 years old from a diversity of ethnic backgrounds with a confirmed diagnosis of HbSS. This study was approved by the Albert Einstein College of Medicine Internal Review Board (CCI protocol #2008–201). Written and signed informed consent was obtained from patients or their guardians. Control peripheral blood samples were provided as discarded whole blood units from the New York Blood Center (New York, NY). Peripheral blood mononuclear cells were isolated with Histopaque-1077 (Sigma-Aldrich; St. Louis, MO, USA) following vendor’s protocol. CD34^+^ cells were isolated using the Positive Selection EasySep Human CD34^+^ Selection Kit according to vendor’s protocol (StemCell Technologies; Vancouver, BC, Canada) Bone marrow aspirates enriched for CD34^+^ were commercially obtained (StemCell Technologies) from eight unrelated, healthy female donors of different ethnic backgrounds. Donors with a self-reported history of hematologic disease or disorders were excluded.

### Cell Lines

AFT024 mouse fetal liver [41] cell line was obtained from ATCC and grown according to ATCC recommendations. When confluent, cells were irradiated at 12000rad and plated to be 90% confluent on 0.1% gelatinized plates. Plates were incubated at 37°C overnight before adding progenitor cells.

### Isolation of Common Myeloid Progenitors from Bone Marrow

CD34^+^ enriched cells from bone marrow mononuclear cells were stained with monoclonal human specific antibodies for phycoerythrin (PE)-Cy5-conjugated lineage markers: CD2 (RPA-2.10), CD3(UCHT2), CD10(CB-CALLA), CD19(SJ25-C1), CD20(2H7) (e-bioscience; San Diego, CA; USA); CD4(S3.5), CD7(6b7), CD8(3B5), CD14(TUK4), CD56(MEM-188), CD235a(CLB-ery-1(AME-1) (Life Technologies; Grand Island, NY, USA) and human specific antibodies CD34-APC(581/CD34 class III epitope) and CD123-PE(6H6) (BD Bioscience, San Jose, CA; USA); CD38- PE-Cy7(HIT-2) (e-bioscience); CD45RA- FITC(MEM-56) (Life Technologies) were used to isolate Common Myeloid Progenitor cells (Lin^-^, CD34^+^, CD38^+^, CD45RA^-^, CD123^low^) (adapted from [42]. CMPs were directly sorted as single cells into 96-well round tissue culture treated plates (BD Biosciences) prepared with irradiated AFT024 cells as described above. Cells were sorted on FACSAria (BD Biosciences) using FACSDiva software (BD Biosciences). Compensations were adjusted with the use of beads (Life Technologies) and unstained CD34^+^ bone marrow was used as negative controls to establish gating strategy (Invitrogen; Carlsbad, CA, USA).

### Growth Conditions

Single cell common myeloid progenitors were grown in erythrocyte expansion and differentiation culture conditions adapted from [43]. Briefly, serum-free basal media StemSpan SFEM (Stem Cell Technologies) with 1% Penicillin-Streptomycin was supplemented with a two-phase cytokine cocktail. In the first 7 days, 10^–6^ M hydrocortisone (Sigma-Aldrich), 13 ng/mL IL3 (PeproTech; Rocky Hill, NJ; USA), 13 ng/mL BMP4 (R&D Systems, Inc.; Minneapolis, MN; USA), 33 ng/mL FLT3L (Prospec; East Brunswick, NJ, USA), 100 ng/mL SCF (Prospec), and 2.7 U/mL EPO (Amgen; Thousand Oaks, CA, USA) supplemented the media. In the second phase, 10^–6^ M hydrocortisone (Sigma-Aldrich), 13 ng/mL IL3 (PeproTech), 13 ng/mL BMP4 (R&D Systems, Inc.), 40 ng/mL SCF (Prospec), 3.3 U/mL EPO (Amgen) and 40 ng/mL IGF1 (Biomedical Technologies; Ward Hill, MA, USA) supplemented the media for the next 7 days. Media was refreshed every 3–4 days in both phases. At day 14, when cells reached the basophilic erythroblast stage of differentiation, experiments were terminated and RNA or protein collected. A constant dose of 15 μmol/liter of HU (Sigma-Aldrich) was titrated in serum-free conditions to achieve a modest HbF increase with admissible cellular viability for downstream analysis. This concentration was maintained throughout the experiment starting from day 4 of single cell experiments. Erythroid developmental stage was confirmed by CD235a and CD71 expression.

### Analysis of Globin Expression

Protein levels were assessed using reverse phase High Performance Liquid Chromatography with Vydac/Grace C4 or Proto300 C4 columns optimized for hemoglobin using a linear gradient of acetonitrile with trifluoroacetic acid. Samples were prepared as described [44]. Briefly, collected cells were washed three times with calcium/magnesium free PBS and lysed in water by 3 freeze-thaw cycles between liquid nitrogen and 37°C water bath. Percent γ-globin was calculated as a ratio of γ-globin to all β-like globins 100*(Gγ+Aγ)/(β+Gγ+Aγ).

Globin expression was analyzed by quantitative real-time (qRT) PCR. Total RNA from cells was extracted using RNeasy Kits (QIAGEN; Valencia, CA, USA) and cDNA synthesized using SuperScript II First-Strand Synthesis System (Life Technologies). qRT-PCR was performed with a one-step SYBR-Green RT-PCR kit (QIAGEN). Primers used were designed in PearlPrimer (Owen Marshall) or with the help of PrimerBank [45]. Primer sequences listed (S1 Fig).

All PCR reactions were performed in triplicates. Result were normalized to GAPDH expression using the ΔCts methods. All qRT-PCR reaction (reverse transcription and PCR reaction) were repeated at least three times on three different days.

The % γ-globin expression was calculated as

100/(1+(1/( fold difference γ/ fold difference β))

Fold differences relative to GAPDH were calculated as 2^^(-ΔCt)^.

Threshold cutoffs for low vs high γ-globin expressing baso-E progeny were below 20% and above 70% γ-globin respectively.

### Analysis Software/Statistical Programs

Statistical significance determined by GraphPad Prism 6.0 (GraphPad Software Inc; La Jolla, CA, USA). Statistically significant results were based on triplicate independent qRT-PCRs with a standard deviation less than 0.5 of triplicates per individual plate. The unpaired Student’s t-test was utilized with statistically significant P values <0.05.

## Results

### γ**-globin expression is highly variable between progenitor populations and donors**

**Fig 1:**
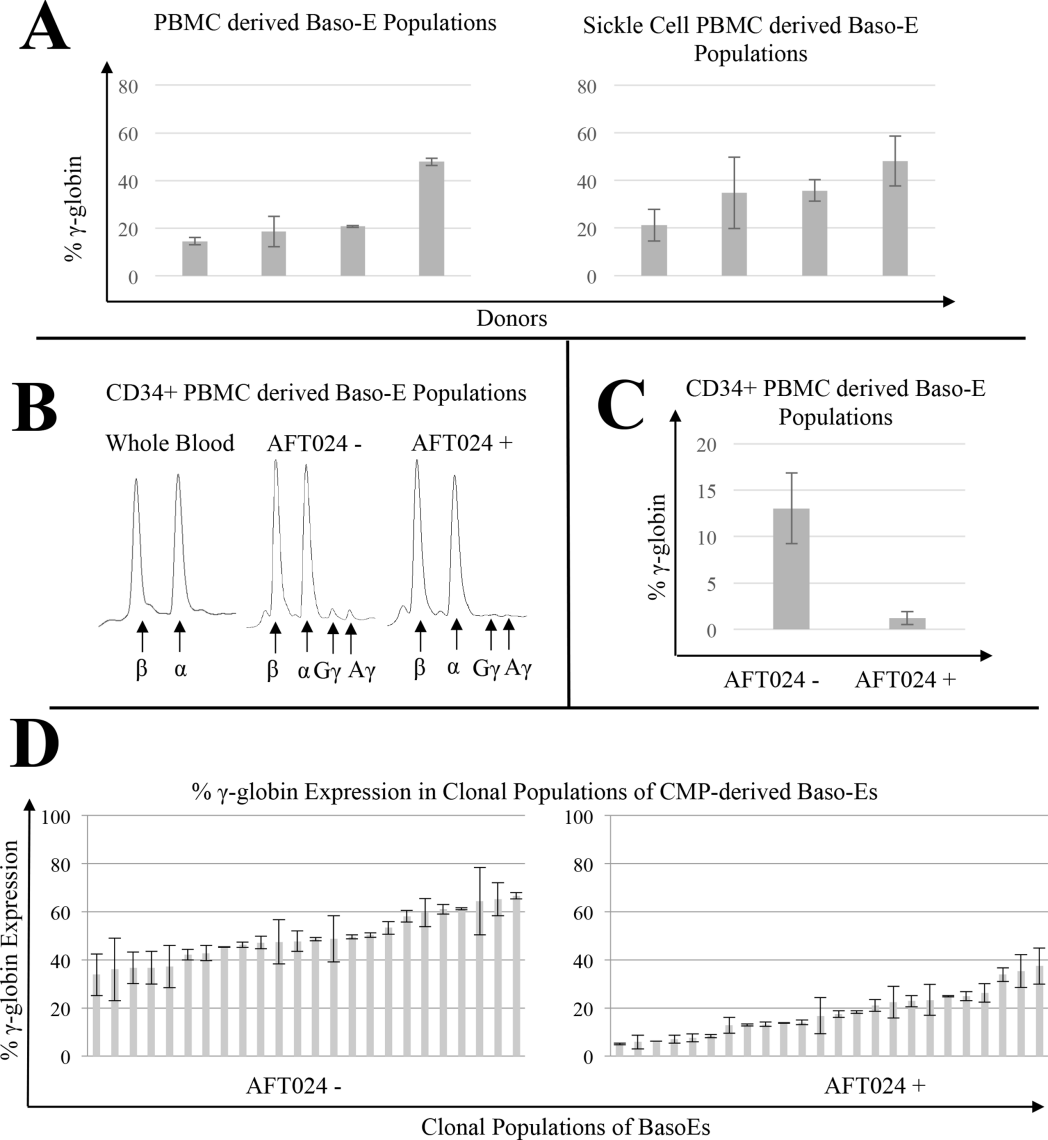
Inter and intra-individual variation in γ-globin expression in baso-Es produced in the presence or absence of AFT024. (A) Variation in HbF levels are recapitulated in culture. Peripheral blood mononuclear cells (PBMCs) from four control donors (left panel) and four sickle cell donors (right panel) were differentiated *in vitro* into populations of baso-Es and globin expression was determined by HPLC. Baso-Es were collected on day 14 of culture system. Percent γ-globin was calculated as ((100*(Gγ + Aγ)/(Gγ + Aγ + β)). Error bars represent standard deviation in γ-globin levels over independent biological replicates. (B) Co-culture of CD34+ cells with AFT024 stromal cells decrease background γ-globin expression. CD34^+^ PBMCs from two control donors were isolated, split into two fractions and differentiated into baso-Es *in vitro* in the presence or absence of mouse embryonic liver stromal cells (AFT024). On day 14, baso-Es were collected for HPLC analysis. Representative chromatograms of globin expression obtained by HPLC analysis from circulating red blood cells *in vivo* (left chromatograms), day 14 baso-E populations derived *in vitro* from CD34^+^ PBMCs without AFT024 stromal cell co-culture (middle chromatogram) or with AFT024 co-culture (right chromatogram). (C) Histogram illustrating the HPLC quantification of the effect of AFT024 on γ-globin expression. Average percent γ-globin shown, error bars represent percent γ-globin variation between donors. (D) Clonal population of baso-E exhibit large variation in γ-globin expression. Individual Common Myeloid Progenitors (CMPs) were isolated from a control bone marrow (BM) donor and placed in culture with or without AFT024 cells to generate clonal populations of baso-Es. After 14 days of culture, the percent of γ-globin expression (γ/γ+β) was determined by qRT-PCR analysis. Error bars represent the standard deviation in average γ-globin expression between replicate rounds of qRT-PCR.

Variation in endogenous γ-globin expression has been associated with several polymorphisms that result in inter-person heterogeneity of HbF before and after treatment with HU [11, 12]. We hypothesized that a serum-free, multi-phase liquid culture experimental system previously defined by our lab and others to enhance adult erythropoiesis could be adapted to preserve this heterogeneity *in vitro* in primary cells isolated from unrelated and ethnically diverse blood donors. The culture system allows for the expansion and differentiation of CD34^+^ hematopoietic progenitors into baso-Es in 14 days [43]. At this developmental stage, high-performance liquid chromatography (HPLC) can be performed to analyze globin expression. We show that peripheral blood mononuclear cells (PBMCs) isolated from both non-sickle and sickle cell donors produce baso-Es populations that preserve inter-donor heterogeneity in HbF levels (Fig 1A and 1B).

In an effort to mimic physiologic baseline γ-globin expression, we modified our culture system by co-culturing the cells on AFT024 mouse embryonic liver stromal cells [41] because it was recently demonstrated that erythroid progenitors derived from baboon BM co-cultured with AFT024 express γ-globin close to physiologic levels [46]. HPLC analysis of baso-Es derived from human BM produced in these *in vitro* conditions express a level of γ-globin nearly equivalent to physiologic expression in contrast to the control without co-culture (Fig 1C and 1D).

Variation in HbF levels observed in biological replicate experiments with small numbers of seed cells prompted us to further refine the culture system. We aimed to (1) standardize the differentiation stage of our starting progenitor population isolated from donor samples to control for HbF variations which might arise from kinetic changes in steady-state hematopoiesis in the donors [47]; and (2) perform experiments on clonally derived cell populations to reveal differences in HbF potential among intra-donor progenitor populations [38].

To accomplish these aims, CMPs expressing a committed lineage-/CD34+/CD38+/CD123low/CD45RA- marker phenotype from healthy bone marrow (BM) donors were sorted directly into 96-well plates at a concentration of one cell per well. After 14 days of culture, the baso-E clones derived from the single cells were collected, RNA was extracted and qRT-PCRs were performed to examine γ-globin expression which reliably correlates to HbF expression [14]. Clonal baso-Es derived from single CMPs showed an intra-donor γ-globin heterogeneity which is preserved even after background γ-globin adjustments with the use of AFT024 co-cultures (Fig 1D). Expression of γ-globin ranged from about 30 to 65% (average = 49.4% +/- 4.02(SD)) and from 5% to 38% (average = 17.97% +/- 2.44 (SD)) in AFT024- and AFT024+ conditions respectively.

### Characterization of γ-globin expression variability in clonal sibling baso-E populations

**Fig 2:**
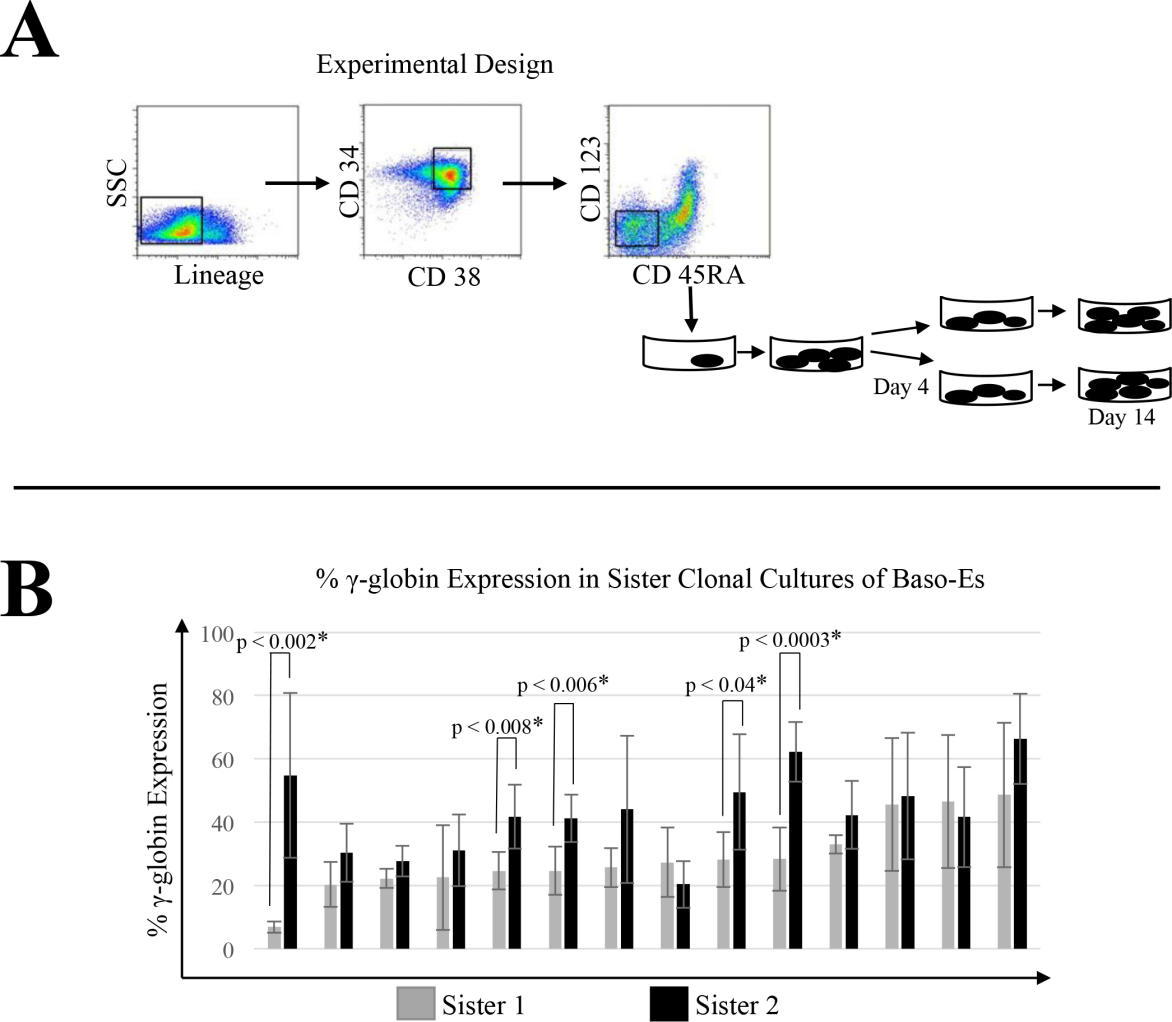
Characterization of γ-globin expression variability in clonal sibling baso-E populations. (A) Experimental design. BM CMPs (box) from control individuals were directly sorted into AFT024 coated 96-well plates at a concentration of one cell per well. After 4 days of culture, progeny from a single CMP were divided into two sister wells and allowed to differentiate in parallel for 10 additional days. RNA from these clonal sibling baso-E populations was then collected for globin analysis. (B) Levels of γ-globin expression are not determined at the CMP level. Percent γ-globin expression was determined by qRT-PCR analysis. Error bars represent the standard deviation between at least three independent cDNA and qRT-PCR replicates. Student’s t-test was used to determine statistically significant differences in γ-globin expression between sister cultures. Levels of γ-globin expression was significantly different in 5 out of 14 sister cultures tested.

In an attempt to prospectively isolate hematopoietic progenitors already committed to either a high or low γ-globin expression program in their more mature progeny, we performed experiments where daughter cells of single BM CMPs were divided into two separate sister wells and allowed to differentiate in parallel for 10 days prior to hemoglobin analysis by qRT-PCR (Fig 2A). The level of γ-globin expression in the sister cultures varied between 5% and 60%. We found that baso-Es expressed significantly different amounts of γ-globin in 5 out 14 sister clonal culture tested (Fig 2B) suggesting that the epigenetic programs for globin gene expression is not defined at the CMP stage of differentiation in control individuals.

### γ-globin expression in response to HU in clonal cultures of baso-Es generated from BM CMPs is variable

**Fig 3:**
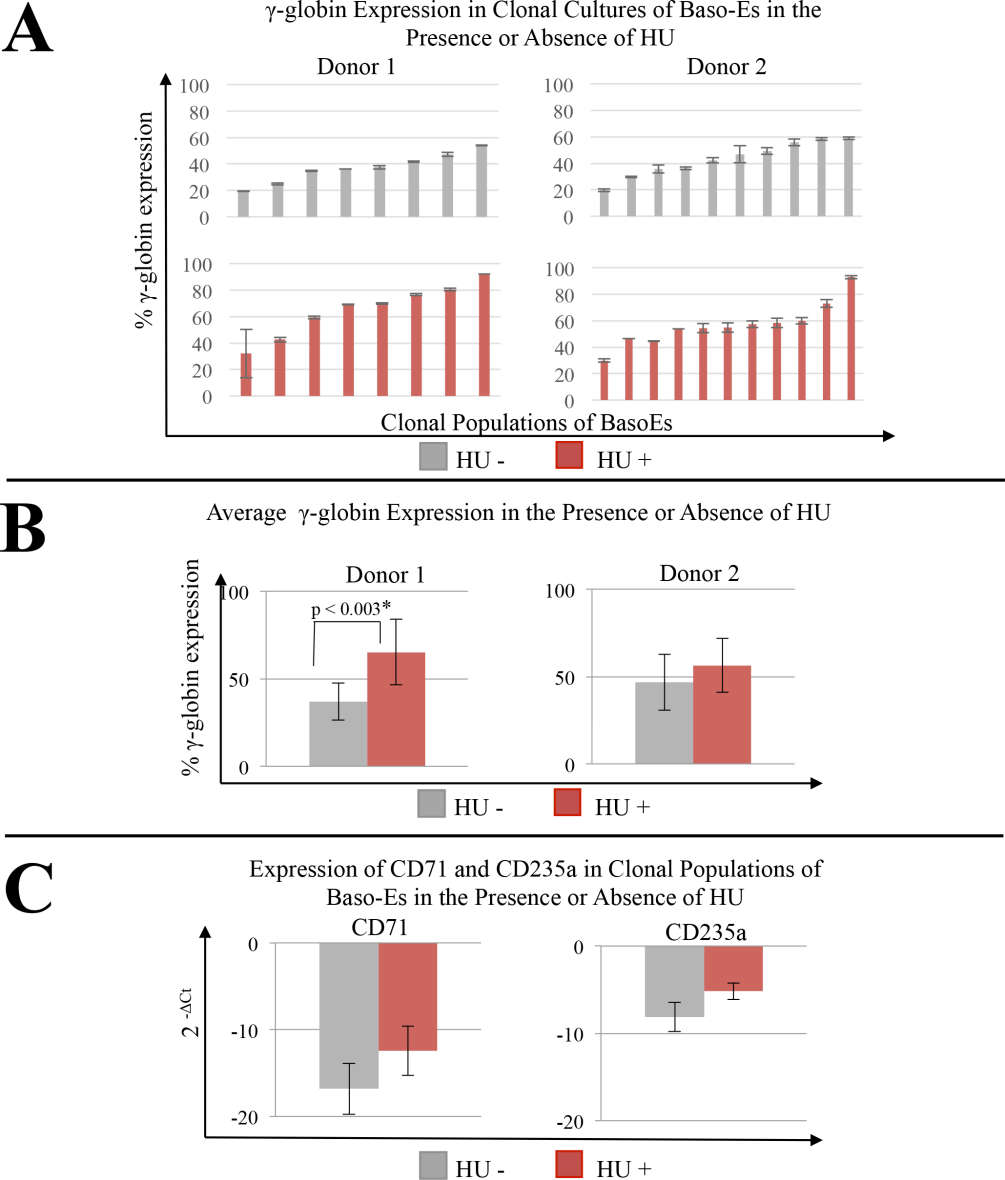
Variability of γ-globin expression in response to HU in clonal cultures of baso-E generated from BM CMPs. (A) Clonal populations of baso-E generated from unicellular BM CMPs from two, unrelated female non-sickle donors (donor 1; donor 2) were generated by co-cultured on AFT024 with or without 15uM HU for 14 days. RNA was extracted and qRT-PCR were performed. Expression of γ-globin expression was calculated as 100*γ/(γ+β). Error bars represent the standard deviation of at least three replicates of the reverse-transcription and qRT-PCR reactions, clonal averages shown. Intra-donor variation between clonal populations is shown at baseline (top panel) and after treatment with HU (bottom panel). (B) Intra-donor γ-globin expression from pooled baseline or HU dosed clones. Error bars represent standard error between clones in dose status group. Student’s t-test was used to determine significant differences in intra-donor γ-globin expression in response to HU. Values p<0.05 are denoted. (C) Intra-donor pooled clones by dose status were evaluated for expression of markers for erythroid differentiation. Student’s t-test with p<0.05 was used to determine significance.

To characterize the response to HU in our clonal progeny assay in a genetically controlled intra-donor setting, we FACS sorted single BM CMPs and added HU to half the wells. γ-globin expression of baso-E clones demonstrated, similar to baseline studies, that an inter-donor and intra-donor heterogeneity exists (Fig 3A). To assess inter-donor response to HU, clones from the same donors were pooled into their dose respective groups and average expression of γ-globin in the presence or absence of induction with HU was compared (Fig 3B). To determine if HU affected the kinetics of erythroblast maturation in our cultured cell system, we compared expression of CD235a and CD71 between baseline and HU conditions. No statistically significant differences were found (Fig 3C).

**Fig 4:**
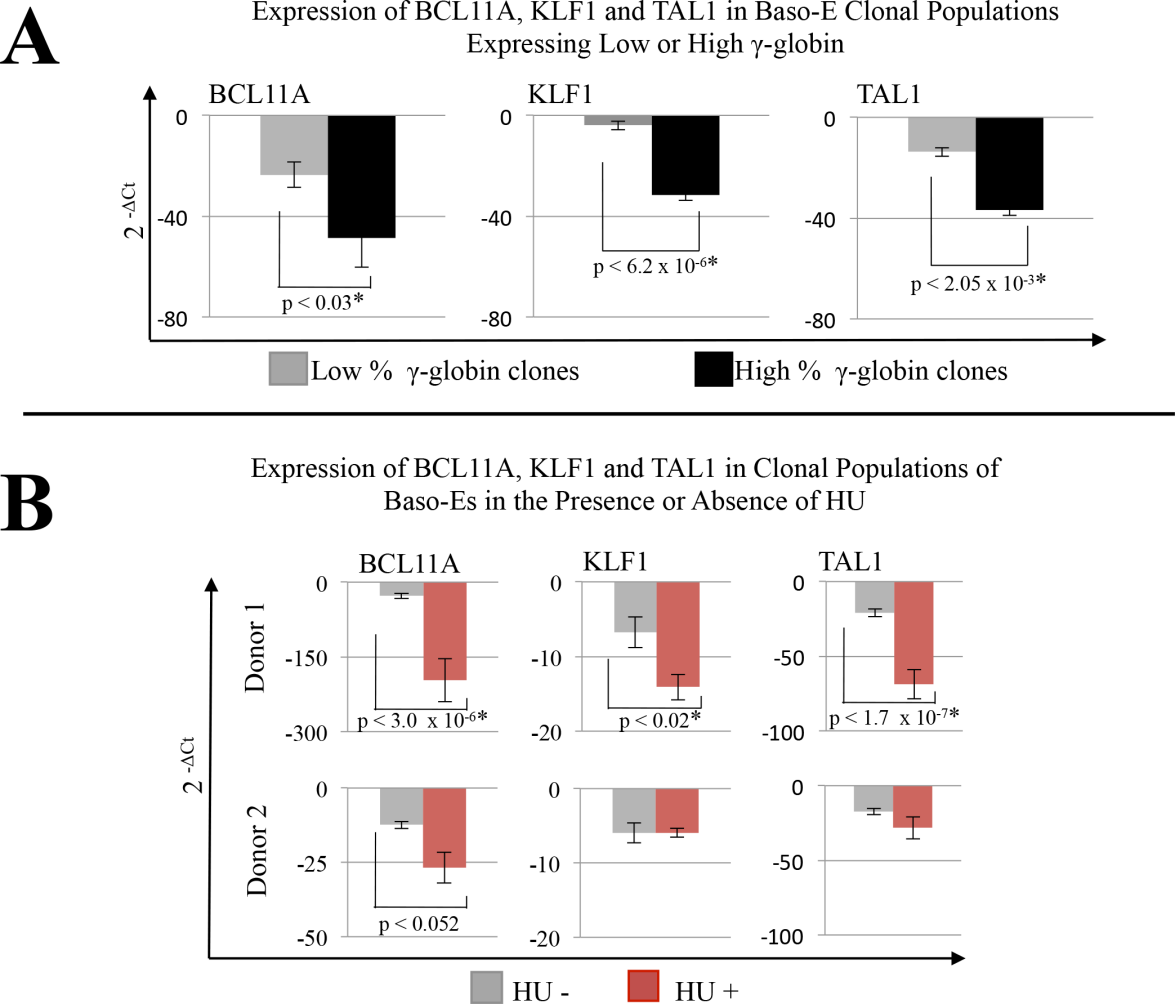
Gene expression analysis in intra-donor unicellular BM CMP derived baso-E progeny. (A) Intra-donor clonal cultures of baso-Es generated in the presence of AFT024 were assigned into groups based on low and high levels of γ-globin expression. qRT-PCRs for select genes were performed. Histograms represent the average fold differences in gene expression relative to GAPDH calculated as 2^^(-ΔCt)^. Error bars represent the standard error of the mean between independent clones pooled into expression group. Statistical significance was determined using student’s t-test with significant values p<0.05 denoted. (B) Clonal cultures of baso-Es from two non-sickle donors (donor 1 and 2 from Fig 3) were co-cultured on AFT024 with or without 15μM HU. qRT-PCRs for select genes were performed on individual clones in triplicate. Fold differences relative to GAPDH calculated as above. Histograms represent the average expression of the pooled clones and error bars represent the standard error between individual clones in dose status group. Statistical significance determined as above.

To further explore the gene program differences in intra-donor clones that might explain variation in baseline γ-globin expression level, qRT-PCR was performed on candidate genes that have previously been implicated in the regulation of erythropoiesis and globin expression. Analysis of 10 to 15 clonal cultures with a panel of PCR primers including BCL11A, KLF1, TAL1, GATA1, GATA2, SOX6, CD71 and CD235a revealed statistically significant reduced levels in expression in the high γ-globin cultures compared to the low γ-globin cultures for only three genes: BCL11A, KLF1 and TAL1 (Fig 4A and S2 Fig). The average fold differences in gene expression relative to GAPDH (2^^(-ΔCt)^) in the low γ-globin clones were -23.6 +/- 5.0 (mean +/- standard error) for BCL11A, -3.9 +/- 0.6 for KLF1, and -13.7 +/- 3.4 for TAL1. In the high γ-globin clones, the average fold differences were -48.6 +/- 11.7 for BCL11A, -31.5 +/- 9.1 for KLF1 and -36.6 +/- 3.6 for TAL1.

To determine the changes in expression of these specific transcription factors with HU mediated induction of γ-globin, we performed qRT-PCR on clonal cultures derived from two healthy bone marrow donors. We found these donors differed in their response to HU *in vitro*. Donor 1 responded with a statistically significant increase in γ-globin and significant decreases in BCL11A, TAL1 and KLF1. In comparison, donor 2 failed to significantly respond to induction of γ-globin and also failed to reach statistically significant decreases in expression of all three genes (Fig 4B). The average fold differences in gene expression relative to GAPDH (2^^(-ΔCt)^) in donor 1 baseline clonal populations were -27.91 +/- 4.7 for BLC11A, -6.7 +/- 2.0 for KLF1 and -21.0 +/- 2.6 for TAL1; and the average fold differences in the presence of HU were -197.1 +/- 43.3 for BCL11A, -14.09 +/- 1.7 for KLF1, and -68.8 +/- 9.9 for TAL1.

**Fig 5:**
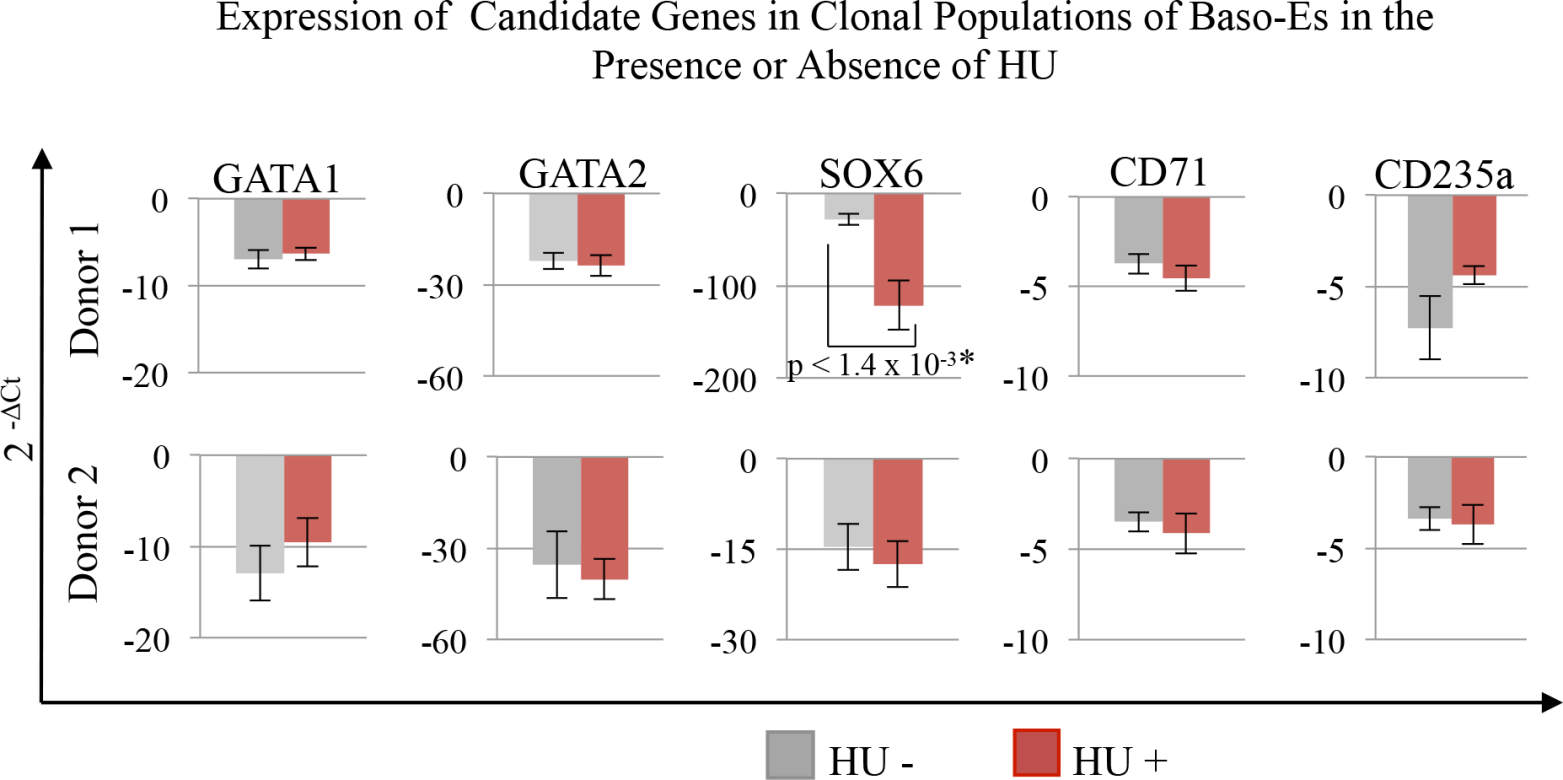
Expression of candidate genes in clonal populations of baso-Es in the presence or absence of HU. Clonal cultures of BM CMP derived baso-Es from two non-sickle donors (donor 1 and 2 from Fig 3) co-cultured on AFT024 with or without 15μM HU. qRT-PCRs for candidate genes were performed in triplicate. Fold differences relative to GAPDH were calculated as 2^^(-ΔCt)^. Histograms represent the average expression of the pooled clones and error bars represent the standard error between individual clones in dose status group.

Other candidate genes tested revealed that response to HU induction was also associated with decreased expression of SOX6 (Fig 5). GATA1, GATA2, CD235a and CD71 expression indicate no statistically significant differences in expression between baseline and HU conditions in either the responding or non-responding donor (Fig 5).

## Discussion

Using baso-Es derived *in vitro* from CD34^+^ cells enriched from unrelated, healthy human bone marrow donors, we found that clonal cultures derived from individual CMPs expressed considerable heterogeneity in the level of expression of HbF, even when daughter cells derived from a single CMP were cultured and analyzed in parallel. This finding suggests that the level of γ-globin expression in baso-Es may be determined late during the differentiation process and that a single CMP can give rise to baso-Es that express either low or high γ-globin levels. Q-RT-PCR analysis of multiple baso-E clones derived from the same donor revealed that high levels of γ-globin expression were associated with decreased expression of the major transcriptional regulators BCL11A, KLF1 and TAL1.

The mechanisms that control the level of expression of these three transcription factors in individual baso-Es and that ultimately determine the levels of HbF expression are still under investigation. Our culture system is highly standardized, serum-free and includes only recombinant cytokines. Therefore, it seems unlikely that the variations in γ-globin expression are caused by changes in the environment, particularly because the variations in globin expression were observed in multiple independent experiments using multiple donors (Fig 1). Additionally, similar levels of CD71 and CD235a among the clones indicate that the differences in gene programs observed cannot be explained by differences in maturation levels of the baso-Es because they are at similar stages of differentiation. The most likely explanation is that expression of the β- and γ-globin genes is inherently unstable and that stochastic processes controlling the levels of expression of BCL11A, KLF1 and TAL1 late in erythroid differentiation determine whether β- or γ-globin will be predominantly expressed.

Comparison of clonal cultures derived from individual CMPs grown in the presence or absence of HU revealed that a decrease in expression of the three transcription factors BCL11A, KLF1 and TAL1 is also associated with higher levels of γ-globin expression. This observation suggests that the same transcription factor networks responsible for high γ-globin expression in a subset of normal baso-Es play a role in induction of γ-globin by HU. This conclusion is supported by the observation in donor 2 where failure to respond to HU was associated with a lack of expression change in these factors.

Since lack of CD71 and CD235a expression changes with HU indicate the kinetics of erythroid differentiation was not markedly altered with the addition of HU (Fig 5), it is likely that HU acts by resetting the basal program of expression for these three factors (BCL11A, KLF1, TAL1) to a lower level. Lowering basal expression would increase the likelihood of γ-globin expression but continue to preserve the γ-globin variability that we observed among individual baso-Es grown in the absence of this drug (S2 Fig). Clinical response to HU may, therefore, depend on resetting the basal expression of these factors to levels low enough to favor γ-globin expression over β-globin in a critical number of cells and may explain why gene expression studies on total populations of erythroid cells have yet to implicate all three factors in the HU response.

Several known interactions between TAL1, KLF1, BCL11A can help explain, in molecular terms, the high γ-globin expression in baso-Es that occurs spontaneously or after induction by HU.

The first interaction is through BCL11A which negatively regulates γ-globin expression. TAL1 has recently been shown to bind to an intronic enhancer in BCL11A [48]. Bauer et al have shown that lower binding of TAL1 at this site in the presence of the minor T allele was associated with lower BCL11A and high HbF expression suggesting that TAL1 might negatively regulate HbF by positively regulating BCL11A.

The second interaction is through TAL1 and the LCR. TAL1 is a DNA-binding component of the pentameric LDB1 complex and has been shown to be indispensable to long range interactions between the LCR and globin genes in K562 cells [49]. Decreased expression of TAL1 might therefore interrupt looping of the LCR to the β-globin promoter causing a decrease in its expression and providing a competitive advantage for other complexes to induce γ-globin expression.

The third interaction is through KLF1, a known inducer of BCL11A [27, 31]. Two lines of evidence suggest that TAL1 might regulate KLF1 both directly and indirectly. TAL1 has been shown to bind to the KLF1 promoter (ENCODE consortium) and to the intergenic enhancer binding site of the MYB gene, itself an activator of KLF1 [50, 51]. A 3bp indel in the regulatory region of MYB alters the spatial configuration of the TAL1/GATA1 enhancer binding site and results in a decrease in MYB expression associated with high HbF [52] suggesting TAL1 may indirectly regulate KLF1 through this mechanism.

Taken together, these observations suggest that in low HbF cells, γ-globin expression is low because BCL11A expression is high because TAL1 and KLF1 are high. High levels of BCL11A and TAL1 favor looping of the LCR to the β-globin gene promoter enhancing its expression. [26, 34]

In high HbF cells, BCL11A is low because TAL1 and KLF1 are low and the γ-globin gene promoters can compete for contact with the LCR.

In the presence of HU, we observe that the same transcription factor network is involved with γ-globin induction along with a decrease in SOX6 expression suggesting the β-globin locus chromatin has been configured to favor γ-globin expression [26]. This decrease in SOX6 expression supports the findings by Flanagan et al in sickle cell patients but is in opposition to Pourfarzad et al results in β-thalassemia patients. Experiments to confirm our results in sickle cell baso-Es remain to be performed but are encourage by corroboration of SOX6 and BCL11A data with Flanagan et al.

The major conclusions of these studies is that production of baso-Es that express high HbF, both spontaneously in healthy individuals during steady state erythropoiesis and after induction with HU, is controlled by the same three transcription factors BCL11A, KLF1 and TAL1. It is unclear how HU controls expression of these three factors and of SOX6. Whether expression of these factors during late erythroid differentiation or after induction with HU is controlled by a single gene or by meta-stable erythroid networks differing by the expression of many genes established in a stochastic manner requires additional research.

## Supporting Information

S1 Fig Primer Sequences. (TIF)

S2 Fig Expression of candidate genes in clonal populations of baso-Es as a function of γ-globin expression. (TIF)
