# Supplementary figures and images for: Variation in Gamma-Globin Expression before and after Induction with Hydroxyurea Associated with BCL11A, KLF1 and TAL1
Source: PLoS One. 2015 Jun 8;10(6):e0129431. doi: 10.1371/journal.pone.0129431 (PMC4459969; doi:10.1371/journal.pone.0129431)

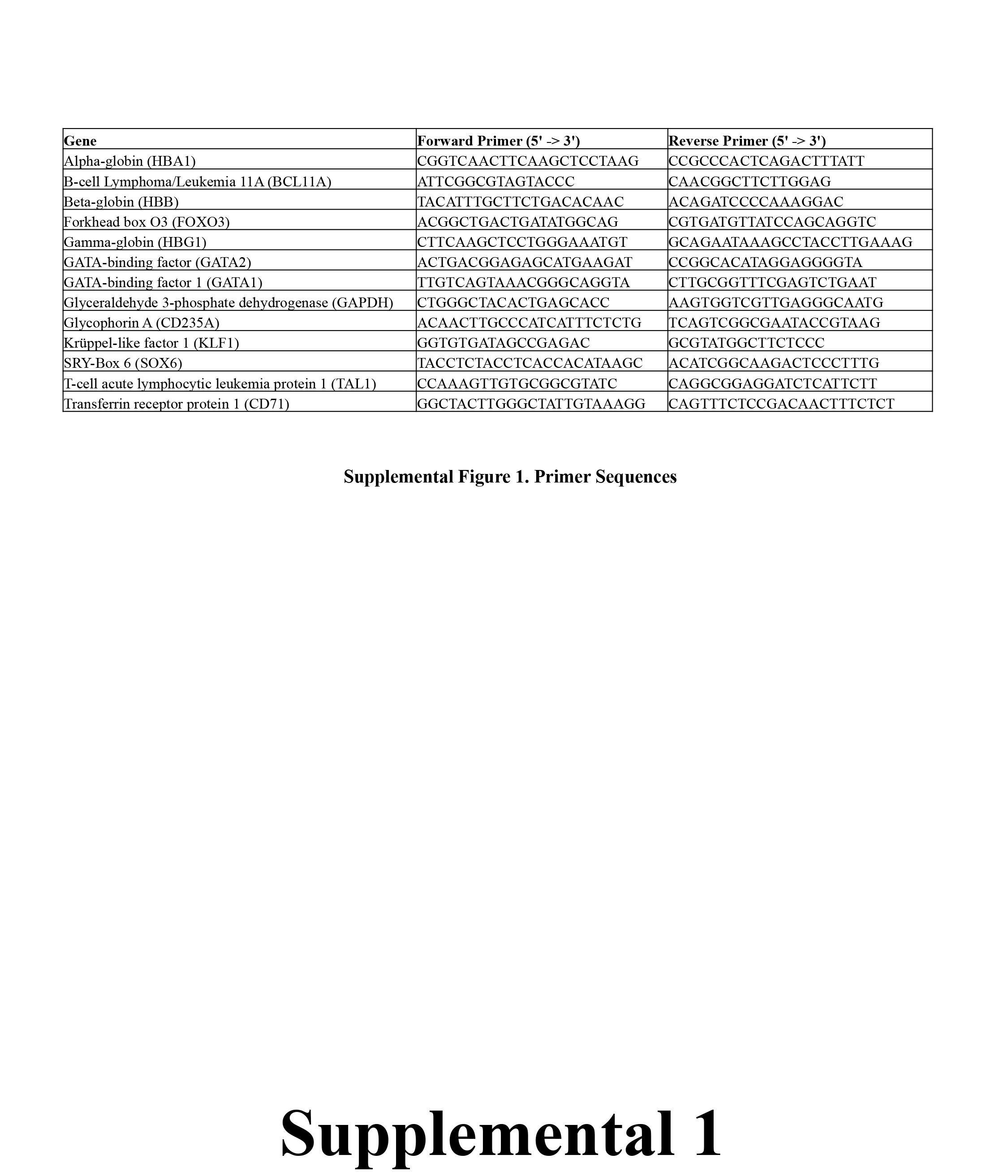

Supplement: S1 Fig — (TIF) [file pone.0129431.s001.tif]

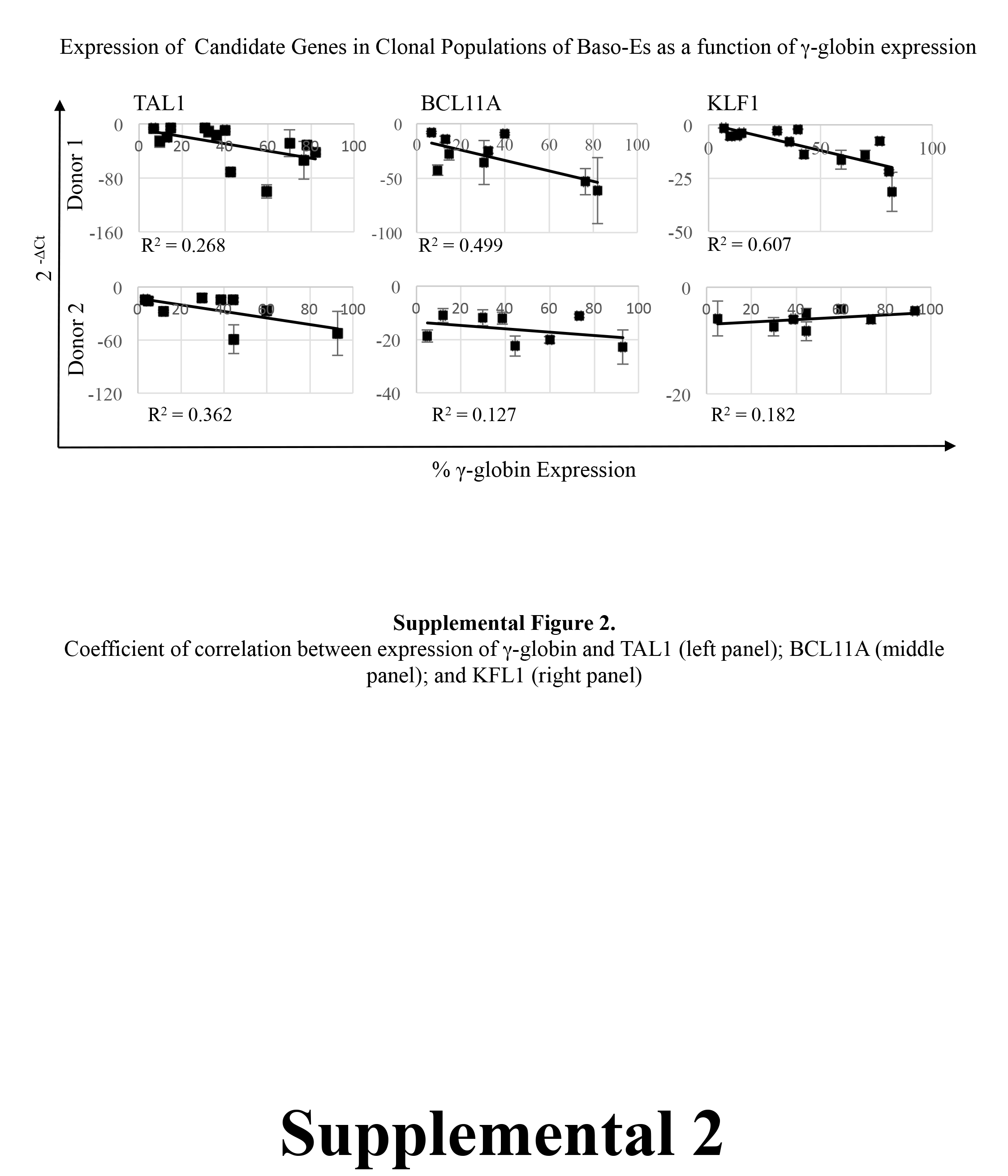

Supplement: S2 Fig — (TIF) [file pone.0129431.s002.tif]
